# Progress in the last decade in our understanding of primary progressive aphasia

**DOI:** 10.4103/0972-2327.74255

**Published:** 2010-12

**Authors:** Ellajosyula Ratnavalli

**Affiliations:** Department of Neurology, Manipal Hospital, Bangalore, India

**Keywords:** Alzheimer’s disease, frontotemporal dementia, logopenic aphasia, neuroimaging, nonfluent aphasia, primary progressive aphasia, semantic dementia

## Abstract

Primary progressive aphasia (PPA) is a focal neurodegeneration of the brain affecting the language network. Patients can have isolated language impairment for years without impairment in other areas. PPA is classified as primary progressive nonfluent aphasia (PNFA), semantic dementia (SD), and logopenic aphasia, which have distinct patterns of atrophy on neuroimaging. PNFA and SD are included under frontotemporal lobar degenerations. PNFA patients have effortful speech with agrammatism, which is frequently associated with apraxia of speech and demonstrate atrophy in the left Broca’s area and surrounding region on neuroimaging. Patients with SD have dysnomia with loss of word and object (or face) meaning with asymmetric anterior temporal lobe atrophy. Logopenic aphasics have word finding difficulties with frequent pauses in conversation, intact grammar, and word comprehension but impaired repetition for sentences. The atrophy is predominantly in the left posterior temporal and inferior parietal regions. Recent studies have described several progranulin mutations on chromosome 17 in PNFA. The three clinical syndromes have a less robust relationship to the underlying pathology, which is heterogeneous and includes tauopathy, ubiquitinopathy, Pick’s disease, corticobasal degeneration, progressive supranuclear palsy, and Alzheimer’s disease. Recent studies, however, seem to indicate that a better characterization of the clinical phenotype (apraxic, agrammatic, semantic, logopenic, jargon) increases the predictive value of the underlying pathology. Substantial advances have been made in our understanding of PPAs but developing new biomarkers is essential in making accurate causative diagnoses in individual patients. This is critically important in the development and evaluation of disease-modifying drugs.

## Introduction

Primary progressive aphasia is a slowly progressive language disorder due to a primary neurodegenerative disease of the brain. It was brought to the attention of contemporary literature only a quarter century back but there have been tremendous advances in our understanding of it’s neurolinguistics, neuroanatomy, neuroimaging, pathology, and molecular genetics. Originally described by Pick in 1892, there was renewed interest in this subject with the description of six patients with insidious onset of dysphasia over 5–11 years by Mesulam in 1982.[1] As the patients had no other cognitive deficits he labeled the disorder “slowly progressive aphasia”. Subsequently, the term “primary progressive aphasia” (PPA) was introduced and the neuropsychological features and longitudinal course were reported.[2,3] Snowden and others coined the term “semantic dementia” (SD) to describe patients with fluent aphasia and loss of semantic knowledge.[4] There followed papers on the neuropsychology, neuroimaging, and neuropathology of PPA.[5–7] Consensus criteria for the diagnosis of frontotemporal lobar degeneration (FTLD) were formulated in 1998 and primary nonfluent aphasia (PNFA) and SD were included under the rubric of FTLD along with frontotemporal dementia (FTD).[8] The diagnosis of PPA can be quite challenging and the confusing terminology and classification have made it more so. The earlier papers on PPA emphasized the nonfluent aphasias and their distinction from Alzheimer’s disease (AD).[2,9] However, recent studies are focusing on developing biomarkers for refining the diagnosis using fluorodeoxyglucose positron emission tomography (FDG-PET), voxel-based morphometry (VBM), and other techniques and correlating with molecular genetics and the underlying pathology. This article will review the different clinical syndromes of PPA with emphasis on the recent advances in neuroimaging, genetics, and pathology.

## Genetics and Risk Factors

Vasectomy rates were significantly higher in PPA compared to normal controls (40% vs 16%) in one study.[10] The authors speculated that vasectomy induced immune response to sperm, which shares antigen epitopes with brain. Learning disability may be a risk factor in increasing the selective vulnerability of language network to degeneration in PPA as learning disabilities were significantly more frequent in patients with PPA and their first degree relatives compared to FTD and AD.[11] Hereditary dysphasic disinhibition dementia (HDDD) was initially consideredo to be a tauopathy linked to chromosome 17, but has been found to be FTLD- U (Ubiquitin) and is caused by a missense mutation in the progranulin (*PGRN*) gene.[12,13] Recently, PPA has been described in patients carrying *PGRN* mutation on chromosome 17.[14–17] A recent study found a splicing mutation in the *PGRN* gene (c709-1G>A) in patients with FTD, 24% of whom presented with PNFA. Many of these patients went on to develop corticobasal syndrome (CBS).[18]

## Definition and Classification

Diagnostic criteria for PPA specify that there has to be an insidious onset and progressive language difficulty for at least two years without behavioral changes, memory or visuospatial impairments.[9] Ideomotor apraxia and mild impairments in calculation or copying can be present on testing but activities of daily living should not be affected by any of these cognitive or behavioral changes. Neuroimaging should rule out a stroke or tumor.[9] Some patients may have only dysphasia for 10–14 years before developing impairments in other cognitive functions.[9]

PPA can be classified into three distinct clinical variants based on language profiles, progressive nonfluent aphasia (PNFA), semantic dementia (SD), and the recently characterized logopenic or phonological variant (LPA).[3,8,19,20] These variants are associated with signature patterns of atrophy and glucose hypometabolism in the language network and with different neuropathologies. Though they are distinct clinical syndromes, some patients can show features of more than one variant and there may be an overlap in syndromes over the progressive course of disease. Both SD and PNFA are included under FTLDs.[8] This is justified by the UK researchers as they share similar behavioral changes and there is significant overlap in the pattern of atrophy on neuroimaging and pathology.[21–23]

There are no epidemiological studies on the prevalence of PPA. Depending on the age range and the clinical setting, 5–20% of all dementias have FTD.[24] The prevalence of FTD in the 45–65 years age group is 15/100,000 and is similar to that of AD.[25,26] In the clinic setting, of the FTLDs, SD and PNFA account for 25% each and FTD accounts for 50% of patients.[21–23]

## Progressive Nonfluent Aphasia

Patients with PNFA are usually older than patients with FTD or SD. Age of onset is usually in the early sixties and it appears to be more common in women. The median survival is around 10 years.[22,23,27–29] The consensus criteria for diagnosis of PNFA specify that agrammatism, phonemic paraphasias or anomia should be present along with a nonfluent speech.[8] The speech is effortful, agrammatic, telegraphic and may be associated with stuttering, dysarthria or apraxia of speech (AOS).[9,19,30,31] Single word comprehension is good but syntactic comprehension is impaired. Phonemic paraphasias are noted on naming tasks.[3,7,9,22]

Episodic memory, visuospatial function, attention and executive functions are normal early in the course. Patients are usually independent and have intact social skills. Some patients maintain or even intensify their involvement in complex hobbies even during late stages, when the patient is mute and is unable to communicate.[9]

## Associated Deficits

Acalculia, buccofacial, and ideomotor apraxia may be present. Mild deficits in visuospatial function can also be seen.[9] A few patients may develop features of motor neuron disease late in the course of the disease. Associated mild pyramidal signs on the right side may be present.[7] Speech and language deficits remain isolated for many years but development of a generalized extrapyramidal syndrome compatible with a diagnosis of CBS is common.[32–35]

## Neuroimaging

**Figure 1:**
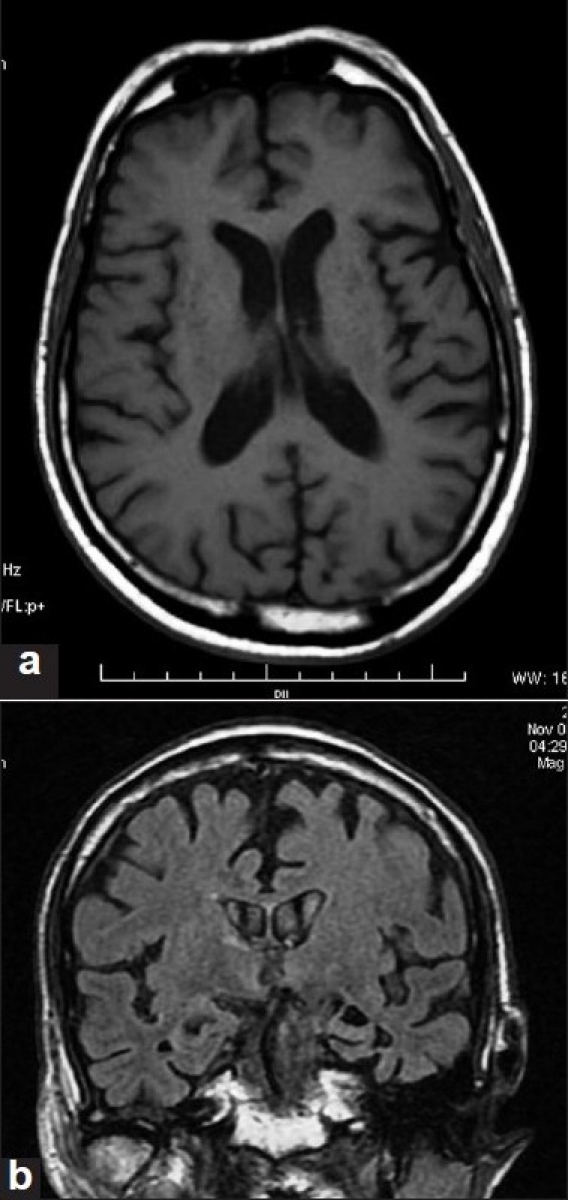
Magnetic resonance imaging brain T1W axial (a) and coronal (b) images showing left perisylvian and temporal lobe atrophy in a 77-yearr-old patient with progressive nonfluent aphasia

There is left perisylvian atrophy on neuroimaging.[9] [Figure 1]. Magnetic resonance imaging using VBM reveals atrophy in the inferior and middle frontal gyri, dorsal motor and premotor cortex, anterior insula, basal ganglia, and supplementary motor area in the left hemisphere.[19,20,30] Selective involvement in specific areas leading to distinct clinical symptoms has been demonstrated using VBM. For instance, patients with pure motor speech deficits show dorsal left premotor and supplementary motor area atrophy, nonfluent aphasics left inferior frontal atrophy, agrammatics atrophy in the left middle frontal and inferior frontal gyri and patients with early mutism left frontal opercular and basal ganglia damage.[30,31,36] These studies also validate that different neural substrates subserve processing of different components of the speech and language. PET studies demonstrate left frontal and anterior insular hypometabolism.[7,37,38]

## Neuropathology

**Figure 2:**
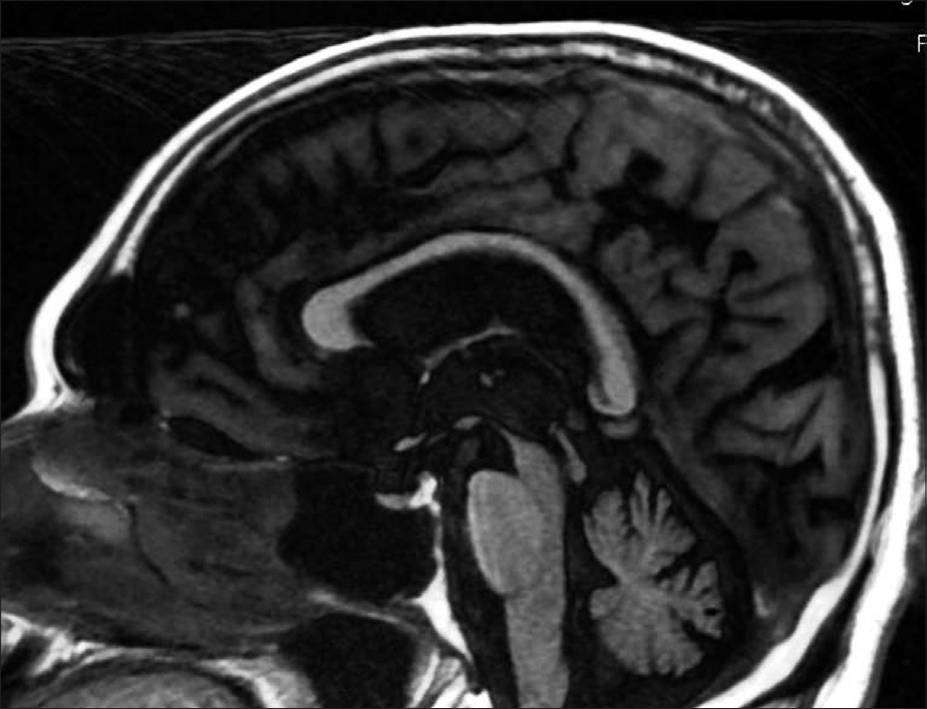
MRI brain T1W sagittal image showing atrophy of midbrain tegmentum – the humming bird sign in a 67-year-old man with progressive nonfluent aphasia and speech apraxia

A number of clinicopathological studies have now documented that tauopathies are the most common underlying cause of PNFA, particularly PSP, CBD, and Pick’s disease.[30,35,39] When AOS is dominant, PSP appears to be common and when AOS occurs with aphasia, CBD pathology is predominant.[30] [Figure 2]. Focal AD pathology is found in as many as 30% of cases.[35,39] Tau-negative, ubiquitin-positive inclusions, and Pick disease pathology are less common and each account for approximately 10% of typical PNFA cases.[35,39]

## Semantic Dementia

The usual age of onset is between 55–70 years with no sex predilection.[21,22] In a recent paper on 100 patients with SD, the mean age at diagnosis was 64.2 years with a range of 40–79 years.[40] The authors emphasized that contrary to expectations, 46% of patients were diagnosed after 65 years and 7 after the age of 75. Family history appears to be less common as compared to FTD and only 2–10% had a family history of early onset dementia or Pick’s disease.[22,40] The 50% survival rate was 12.8 years, more benign than hitherto believed.[40]

Patients with SD present with word finding difficulties or dysnomia. Speech may be fluent but circumlocutory and contains semantic paraphasias.[4,5,21] As word finding difficulties increase, patients substitute the target word with a generic word for instance “animal” for “cat” and “thing” for “pen”.[4,5,21] Single word comprehension is also affected but may not be recognized in the early stages. Sentence comprehension, repetition, and grammar are relatively intact.

Patients show deficits in nonverbal tasks using auditory, visual, and other modalities suggesting a breakdown in conceptual knowledge rather than deficit in language. These aspects can be probed by asking the patients to define words, naming words to definition, pyramids, and palm test and color object decision task.[21,41–43] Patients may not be able to name pictures and also cannot point to the correct picture when named. They cannot describe the nature of the object or its use suggesting a deficit in semantic memory.[21,42,44] Patients have been described with predominant involvement of the right temporal lobe who have prosopagnosia.[45,46]

On neuropsychological testing, working memory, calculations and visuospatial abilities are preserved.[6,47–49] Category fluency is reduced but letter fluency is better. Recent memory is preserved but patients may do poorly on verbal learning tasks because of impaired semantic memory.[47–49] Visual memory is intact. Surface dyslexia and dysgraphia are common.[50]

The widely used consensus criteria for SD also include that associative agnosia and/or prosopagnosia be present. Mesulam believes that there are two distinct groups of patients –fluent aphasics with anomia and impaired word comprehension with involvement of the language network in the left posterior temporo-parietal area but without agnosia and others with fluent aphasia and agnosia with involvement of bilateral inferotemporal-fusiform network in the temporal lobes.[51] Hodges and others suggest that all patients with so called fluent aphasia and anomia evolve into semantic dementia where they exhibit multimodal object recognition deficits. They emphasize that such deficits may be missed early in the course if impact of concept familiarity and typicality of objects are not taken into account. They argue that this is a reflection of the progressive deterioration of an amodal integrative semantic memory system in the rostral temporal lobes rather than involvement of two distinct areas as postulated by Mesulam.[21,52]

## Associated Features

Associated behavioral changes like apathy, clockwatching and interest in jigsaw puzzles, rigidity, repetitive behaviors, lack of empathy and food fads, have been reported.[53–55] A few patients may show amyotrophy very late in the course but usually the neurological examination is normal.[21] Rosen *et al*. (2006) compared the behavioral features of SD with other PNFA and AD and found that eating disorders, aberrant motor behavior and disinhibition were more common in SD and increased in severity with duration of illness.[55]

## Repeat and Pointing Test

This is a simple test which can be done in the clinic to differentiate PNFA from SD. Patient is asked to repeat a multisyllabic noun (usually a tool/vehicle or an animal/bird) and after repeating it, point to its picture among an array of 4–6 semantic and perceptual distractors. Patients with PNFA are impaired on the repetition task but show no deficit on the pointing component of the task. The converse is true of patients with semantic dementia.[56]

## Neuroimaging

There is bilateral though asymmetrical atrophy of the anterior and medial temporal lobes, which is more evident on the coronal MRI especially in the early stages.[57] The polar and perirhinal cortices and the anterior fusiform gyri are particularly atrophic.[19,57,58] There appears to be a rostral-caudal gradient in SD, with anterior portions of the temporal lobes most affected in contrast to AD which has a caudal-rostral gradient. The preserved episodic memory in spite of the atrophy of the medial temporal lobe is striking and one of the explanations is the involvement of posterior cingulate cortex in AD and not FTD which may have an important role in episodic memory.[59,60] The regional atrophy and hypometabolism are closely coupled in SD but not so in AD, where there is extensive hypometabolism in regions that are not obviously atrophic.[61]

## Neuropathology

Reported literature suggests that 70% of patients have tau-negative ubiquitin positive pathology with a TAR DNA binding protein (TDP-43), 20% tau-positive Pick’disease, and 10% have AD pathology.[23,39,62,63] In a recent study of 100 patients with SD, 24 had autopsy – 18 had FTD-U (13/13 TDP-43 positive), 3 had classical tau pathology, and 3 had AD. There were no intranuclear lentiform TDP-43 inclusions in any of the patients.[40]

## Logopenic or Phonological variant of PPA

**Figure 3:**
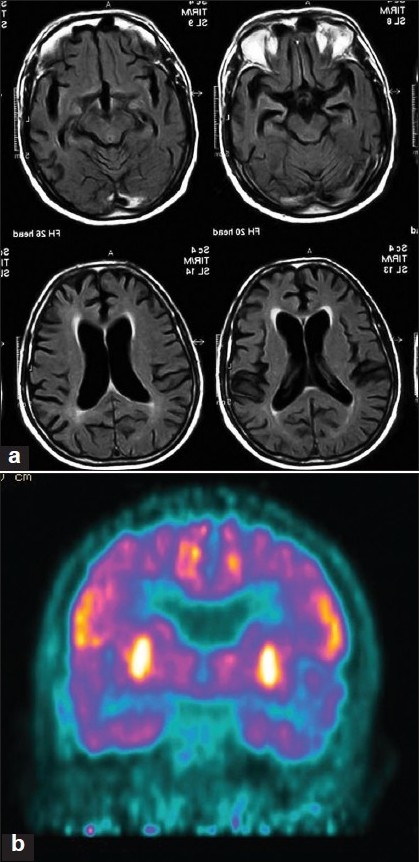
(a) MRI brain T1W axial image showing diffuse atrophy in a 58-year-old man with logopenic aphasia. (b) FDG PET-CT showing hypometabolism in bilateral temporal lobes

This variant has been recently characterized by Gorno-Tempini and others and this accounts for 30% of cases of PPA in their series.[19,20] Patients have slow, hesitant speech with word-finding pauses, which is well articulated and grammatical (differentiating from PNFA) and with good single-word comprehension (differentiating from semantic dementia). There is moderate dysnomia and sentence comprehension is affected. Single word repetition is preserved but sentence repetition is severely impaired. Digit span is reduced. LPA resembles conduction aphasias seen after stroke. Neuroimaging using VBM shows atrophy of the left posterior temporoparietal region with involvement of the left superior, middle temporal gyri, and inferior parietal lobule. [Figure 3]. The cognitive defect appears to be in the phonological loop component of the auditory verbal-working memory. There is a high frequency of apolipoprotein E4 genotype in LPA.[19]

A recent report of patient with features of logopenic PPA who had a mutation in the progranulin gene also had some deficits in grammar, repetition, and semantic memory suggesting that *PGRN* mutation can cause overlapping PPA syndrome.[64]

## Alzheimer’s Disease and Primary Progressive Aphasia

Many patients with AD have language impairment during the course of dementia which is especially evident on neuropsychological testing. A minority can present with predominant aphasia. Initially it was believed that only fluent aphasia can occur in AD.[9] However, clinicopathological studies have shown that AD can cause both PNFA and SD. Recent literature suggests that most patients with AD have logopenic aphasia.[20] Because accurate diagnosis is essential for appropriate therapy, there has been considerable interest in identifying clinical predictors of FTLD versus AD pathology in PPA. On cognitive testing, associated deficits in attention and memory maybe evident, which are more suggestive of AD. Mendez *et al*. (2003) compared 15 patients with PNFA and AD and found that literal paraphasias, stuttering, and decreased speed of utterances in PNFA.[65] A recent study found that AD patients with aphasia had better processing speed than PPA patients and they had temporoparietal atrophy with sparing of the anterior temporal lobe and hippocampi on VBM, in contrast to the FTD group who had anterior and medial temporal lobe atrophy with sparing of the parietal lobe.[66] Involvement of the posterior cingulate or involvement of parietal lobe on structural or functional imaging may also support the diagnosis of AD.[59,61,66]

Some investigators have reported a disproportionately high burden of plaques and tangles in the left temporal and inferior parietal cortex (language areas) in AD presenting as PPA.[39,67] Others, however, do not find a consistent asymmetry or neocortical preponderance of AD pathology in language-related areas in these patients with PPA.[67,68] A recent study of PPA using FDG-PET and ^[11C]-^Pittsburgh compound B imaging reported an association of LPA with diffuse amyloid deposition though there was focal hypometabolism in the left temporoparietal region.[69] Mesulam and his group also did not find a concordance between the AD pathology and the asymmetric atrophy in their recent pathology study of PPAs. They suggest that either the AD in these cases of PPAs is truly atypical or there is a concomitant process that triggers dysphasia.[70]

## Clinicopathological Correlations – Recent Studies

Each PPA syndrome is associated with a specific pattern of atrophy on neuroimaging but appears to have less robust relationship with underlying neuropathology[71] in contrast to a few reports that FTD-U correlates with right temporal lobe atrophy[72] or FTD-T with striatal atrophy.[73] The heterogeneity of pathological picture is possibly the result of different methodology applied to patient population with variability in the use of language tests and variations in the definitions of semantic dementia, fluency, and agrammatism. PNFA was largely and SD exclusively associated with TDP- 43 proteinopathy in a recent study from Manchester.[23] PNFA was associated with FTD-U type 3 and SD with FTD-U type 1. In this study, only 11% PNFA had tauopathy and none of the SD patients had tauopathy in contrast to the earlier literature quoted above. The authors argue that tauopathies reported in PNFA are probably associated with AOS rather than aphasia. They also believe that the SDs included in their study were “pure ” SD without behavioral changes. Behavioral variants of FTD, which are associated with tau pathology and in particular, microtubule-associated tau mutations (*MAPT*), may demonstrate semantic memory impairment during the course of the disease.[74] Predictive value of clinical diagnosis can be improved by subtyping PNFA into agrammatic and logopenic suggested one recent study as most of the agrammatics had FTLD-T and the logopenics had AD.[70] Similar robust clinicopathological correlations are reported by a longitudinal study which investigated 18 patients of PPA over a 15 year period and classified PPA into 5 different types. Patients with pure dysarthria had tau pathology (CBD, PSP, and pick’s disease), agrammatics and typical SD had an ubiquitin positive TDP-43 proteinopathy, jargon and logopenic PPA had AD and atypical SD had CBD or argyrophilic grain disease. In this study, 4 of the 6 agrammatic patients had progranulin mutations.[75]

## Treatment

There are no treatments available at present for PPA. Bromocriptine was used in a double-blind placebo controlled trial in six subjects with PNFA and showed a mild improvement in the mean length of utterances.[76] There is one case report of PPA showing some improvement with the use of oral steroids.[77] The improvement was mild and the patient received steroids for only three months. An open label trial of memantine for 26 weeks in FTD, SD, and PPA patients failed to show significant benefit.[78]

Cholinesterase inhibitors have not been specifically evaluated in PPA-AD but if the patient has aphasia compatible with AD for instance the logopenic type, these can be tried.

Evaluation by speech therapist to improve and explore alternative communicative strategies may be useful. Non therapeutic measures like learning sign language and use of voice synthesizers or laminated cards to communicate may be beneficial. As many patients continue to be independent and active, it is important to explain the disease and the prognosis to the patient and the family to help cope with the impairment better.

## Conclusions

There have been significant advances in our understanding of the PPAs in the last decade. The definition and the subtyping of PPA continue to evolve but refinement in clinical and pathological characterization is required for better diagnosis of the different clinical syndromes. In future besides structural and functional neuroimaging, cerebrospinal fluid and molecular markers may be available for better diagnosis during life. Differentiating the clinicopathological types is important to evaluate new therapeutic strategies in FTLD or to use disease modifying treatments in AD. As PPA is a focal neurodegeneration with a prolonged longitudinal course it offers an excellent opportunity to develop disease modifying therapies to slow or arrest progression.

## References

[1] Mesulam MM (1982). Slowly progressive aphasia without generalized dementia. Ann Neurol.

[2] Mesulam MM (1987). Primary progressive aphasia: Differentiation from Alzheimer’s disease. Ann Neurol.

[3] Weintraub S, Rubin NP, Mesulam MM (1990). Primary progressive aphasia: Longitudinal course, neuropsychological profile, and language features. Arch Neurol.

[4] Snowden JS, Goulding PJ, Neary D (1989). Semantic dementia: A form of circumscribed cerebral atrophy. Behav Neurol.

[5] Hodges JR, Patterson K, Oxbury S, Funnell E (1992). Semantic dementia: Progressive fluent aphasia with temporal lobe atrophy. Brain.

[6] Hodges JR, Patterson K (1996). Nonfluent progressive aphasia and semantic dementia: A comparative neuropsychological study. J Int Neuropsychol Soc.

[7] Turner RS, Kenyon LC, Trojanowski JQ, Gonatas N, Grossman M (1996). Clinical, neuroimaging, and pathologic features of progressive nonfluent aphasia. Ann Neurol.

[8] Neary D, Snowden JS, Gustafson L, Passant U, Stuss D, Black S (1998). Frontotemporal lobar degeneration: A consensus on clinical diagnostic criteria. Neurology.

[9] Mesulam MM (2001). Primary progressive aphasia. Ann Neurol.

[10] Weintraub S, Fahey C, Johnson N, Mesulam MM, Gitelman DR, Weitner BB (2006). Vasectomy in men with primary progressive aphasia. Cogn Behav Neurol.

[11] Rogalski E, Johnson N, Weintraub S, Mesulam M (2008). Increased frequency of learning disability in patients with primary progressive aphasia and their first-degree relatives. Arch Neurol.

[12] Lendon CL, Lynch T, Norton J, McKeel DW, Busfield F, Craddock N (1998). Hereditary dysphasic disinhibition dementia: A frontotemporal dementia linked to 17q21-22. Neurology.

[13] Mukherjee O, Pastor P, Cairns NJ, Chakraverty S, Kauwe JS, Shears S (2006). HDDD2 is a familial frontotemporal lobar degeneration with ubiquitin-positive, tau-negative inclusions caused by a missense mutation in the signal peptide of progranulin. Ann Neurol.

[14] Snowden JS, Pickering-Brown SM, Mackenzie IR, Richardson AM, Varma A, Neary D (2006). Progranulin gene mutations associated with frontotemporal dementia and progressive non-fluent aphasia. Brain.

[15] Mesulam M, Johnson N, Krefft TA, Gass JM, Cannon AD, Adamson JL (2007). Progranulin mutations in primary progressive aphasia: The PPA1 and PPA3 families. Arch Neurol.

[16] Beck J, Rohrer JD, Campbell T, Isaacs A, Morrison KE, Goodall EF (2008). A distinct clinical, neuropsychological and radiological phenotype is associated with progranulin gene mutations in a large UK series. Brain.

[17] Benussi L, Binetti G, Sina E, Gigola L, Bettecken T, Meitinger T (2008). A novel deletion in progranulin gene is associated with FTDP-17 and CBS. Neurobiol Aging.

[18] Moreno F, Indakoetxea B, Barandiaran M, Alzualde A, Gabilondo A, Estanga A (2009). “Frontotemporoparietal” dementia: clinical phenotype associated with the c.709-1G>A *PGRN* mutation. Neurology.

[19] Gorno-Tempini ML, Dronkers NF, Rankin KP, Ogar JM, Phengrasamy L, Rosen HJ (2004). Cognition and anatomy in three variants of primary progressive aphasia. Ann Neurol.

[20] Gorno-Tempini ML, Brambati SM, Ginex V, Ogar J, Dronkers NF, Marcone A (2008). The logopenic /phonological variant of primary progressive aphasia. Neurology.

[21] Hodges JR, Patterson K (2007). Semantic dementia: A unique clinicopathological syndrome. Lancet Neurol.

[22] Snowden JS, Neary D, Mann D (1996). Frontotemporal lobar degeneration: Frontotemporal dementia, progressive aphasia, semantic dementia.

[23] Snowden J, Neary D, Mann D (2007). Frontotemporal lobar degeneration: Clinical and pathological relationships. Arch Neuropathol.

[24] Chow TW, Hodges JR, Dawson KE, Miller BL, Smith V, Mendez MF (2005). Referral patterns for syndromes associated with frontotemporal lobar degeneration. Alzheimer Dis Assoc Disord.

[25] Ratnavalli E, Brayne C, Dawson K, Hodges JR (2002). The prevalence of frontotemporal dementia. Neurology.

[26] Harvey RJ, Skelton-Robinson M, Rossor MN (2003). The prevalence and causes of dementia in people under the age of 65 years. J Neurol Neurosurg Psychiatry.

[27] Hodges JR, Davies R, Xuereb J, Kril J, Halliday G (2003). Survival in frontotemporal dementia. Neurology.

[28] Roberson ED, Hesse JH, Rose KD, Slama H, Johnson JK, Yaffe K (2005). Frontotemporal dementia progresses to death faster than Alzheimer disease. Neurology.

[29] Johnson JK, Diehl J, Mendez MF, Neuhaus J, Shapira JS, Forman M (2005). Frontotemporal lobar degeneration: Demographic characteristics of 353 patients. Arch Neurol.

[30] Josephs KA, Duffy JR, Strand EA, Whitwell JL, Layton KF, Parisi JE (2006). Clinicopathological and imaging correlates of progressive aphasia and apraxia of speech. Brain.

[31] Ogar J, Willock S, Baldo J, Wilkins D, Ludy C, Dronkers N (2006). Clinical and anatomical correlates of apraxia of speech. Brain Lang.

[32] Boeve B, Dickson D, Duffy J, Bartleson J, Trenerry M, Petersen R (2003). Progressive nonfluent aphasia and subsequent aphasic dementia associated with atypical progressive supranuclear palsy pathology. Eur Neurol.

[33] Gorno-Tempini ML, Murray RC, Rankin KP, Weiner MW, Miller BL (2004). Clinical, cognitive and anatomical evolution from nonfluent progressive aphasia to corticobasal syndrome: A case report. Neurocase.

[34] Kertesz A, Martinez-Lage P, Davidson W, Munoz DG (2000). The corticobasal degeneration syndrome overlaps progressive aphasia and frontotemporal dementia. Neurology.

[35] Kertesz A, McMonagle P, Blair M, Davidson W, Munoz DG (2005). The evolution and pathology of frontotemporal dementia. Brain.

[36] Gorno-Tempini ML, Ogar JM, Brambati SM, Wang P, Jeong JH, Rankin KP (2006). Anatomical correlates of early mutism in progressive nonfluent aphasia. Neurology.

[37] Nestor PJ, Graham NL, Fryer TD, Williams GB, Patterson K, Hodges JR (2003). Progressive non-fluent aphasia is associated with hypometabolism centred on the left anterior insula. Brain.

[38] Nestor PJ, Balan K, Cheow HK, Fryer TD, Knibb JA, Xuereb JH (2007). Nuclear imaging can predict pathologic diagnosis in progressive nonfluent aphasia. Neurology.

[39] Knibb JA, Xuereb JH, Patterson K, Hodges JR (2006). Clinical and pathological characterization of progressive aphasia. Ann Neurol.

[40] Hodges JR, Mitchell J, Dawson K, Spillantini MG, Xuereb JH, McMonagle P (2010). Semantic dementia: Demography, familial factors and survival in a consecutive series of 100 cases. Brain.

[41] Howard D, Patterson K (1992). Pyramids and Palm trees: A test of semantic access from pictures and words. Bury St Edmunds, Suffolk.

[42] Bozeat S, Lambon Ralph MA, Patterson K, Garrard P, Hodges JR (2000). Non-verbal semantic impairment in semantic dementia. Neuropsychologia.

[43] Lambon Ralph M, Patterson K, Garrard P, Hodges JR (2003). Semantic dementia with category specificity: A comparative case-series study. Cogn Neuropsychol.

[44] Hodges JR, Bozeat S, Lambon Ralph MA, Patterson K, Spatt J (2000). The role of conceptual knowledge in object use: Evidence from semantic dementia. Brain.

[45] Snowden JS, Thompson JC, Neary D (2004). Knowledge of famous faces and names in semantic dementia. Brain.

[46] Thompson SA, Graham KS, Williams G, Patterson K, Kapur N, Hodges JR (2004). Dissociating person-specific from general semantic knowledge: Roles of the left and right temporal lobes. Neuropsychologia.

[47] Hodges JR, Patterson K, Ward R, Garrard P, Bak T, Perry R (1999). The differentiation of semantic dementia and frontal lobe dementia (temporal and frontal variants of frontotemporal dementia) from early Alzheimer’s disease: A comparative neuropsychological study. Neuropsychology.

[48] Perry RJ, Hodges JR (2000). Differentiating frontal and temporal variant frontotemporal dementia from Alzheimer’s disease. Neurology.

[49] Libon DJ, Xie SX, Moore P, Farmer J, Antani S, McCawley G (2007). Patterns of neuropsychological impairment in frontotemporal dementia. Neurology.

[50] Woollams AM, Ralph MA, Plaut DC, Patterson K (2007). SD-squared: On the association between semantic dementia and surface dyslexia. Psychol Rev.

[51] Mesulam MM, Grossman M, Hillis A, Kertesz A, Weintraub S (2003). The core and halo of primary progressive aphasia and semantic dementia. Ann Neurol.

[52] Adlam AL, Patterson K, Rogers TT, Nestor PJ, Salmond CH, Acosta-Cabronero J (2006). Semantic dementia and fluent primary progressive aphasia: Two sides of the same coin?. Brain.

[53] Bozeat S, Gregory CA, Ralph MA, Hodges JR (2000). Which neuropsychiatric and behavioural features distinguish frontal and temporal variants of frontotemporal dementia from Alzheimer’s disease?. J Neurol Neurosurg Psychiatry.

[54] Snowden JS, Bathgate D, Varma A, Blackshaw A, Gibbons ZC, Neary D (2001). Distinct behavioural profiles in frontotemporal dementia and semantic dementia. J Neurol Neurosurg Psychiatry.

[55] Rosen HJ, Allison SC, Ogar JM, Amici S, Rose K, Dronkers N (2006). Behavioral features in semantic dementia vs other forms of progressive aphasias. Neurology.

[56] Hodges JR, Martinos M, Woollams AM, Patterson K, Adlam AL (2008). Repeat and point: Differentiating semantic dementia from progressive non-fluent aphasia. Cortex.

[57] Kipps CM, Davies RR, Mitchell J, Kril JJ, Halliday GM, Hodges JR (2007). Clinical significance of lobar atrophy in frontotemporal dementia: Application of an MRI visual rating scale. Dement Geriatr Cogn Disord.

[58] Rosen HJ, Gorno-Tempini ML, Goldman WP, Perry RJ, Schuff N, Weiner M (2002). Patterns of brain atrophy in frontotemporal dementia and semantic dementia. Neurology.

[59] Nestor PJ, Fryer TD, Ikeda M, Hodges JR (2003). Retrosplenial cortex-B 29/30-hypometabolism in mild cognitive impairment (prodromal Alzheimer’s disease). Eur J Neurosci.

[60] Nestor PJ, Fryer TD, Hodges JR (2006). Declarative memory impairments in Alzheimer’s disease and semantic dementia. Neuroimage.

[61] Diehl J, Grimmer T, Drzezga A, Riemenschneider M, Forstl H, Kurz A (2004). Cerebral metabolic patterns at early stages of frontotemporal dementia and semantic dementia: A PET study. Neurobiol Aging.

[62] Davies RR, Hodges JR, Kril JJ, Patterson K, Halliday GM, Xuereb JH (2005). The pathological basis of semantic dementia. Brain.

[63] Alladi S, Xuereb J, Bak T, Nestor P, Knibb J, Patterson K (2007). Focal cortical presentations of Alzheimer’s disease. Brain.

[64] Rohrer JD, Crutch SJ, Warrington EK, Warren JD (2010). Progranulin-associated primary progressive aphasia: A distinct phenotype?. Neuropsychologia.

[65] Mendez MF, Clark DG, Shapira JS, Cummings JL (2003). Speech and language in progressive nonfluent aphasia compared with early Alzheimer’s disease. Neurology.

[66] Josephs KA, Whitwell JL, Duffy JR, Vanvoorst WA, Strand EA, Hu WT (2008). Progressive aphasia secondary to Alzheimer disease vs FTLD pathology. Neurology.

[67] Galton CJ, Patterson K, Xuereb JH, Hodges JR (2000). Atypical and typical presentations of Alzheimer’s disease: A clinical, neuropsychological, neuroimaging and pathological study of 13 cases. Brain.

[68] Forman MS, Farmer J, Johnson JK, Clark CM, Arnold SE, Coslett HB (2006). Frontotemporal dementia: Clinicopathological correlations. Ann Neurol.

[69] Rabinovici GD, Jagust WJ, Furst AJ, Ogar JM, Racine CA, Mormino EC (2008). Abeta amyloid and glucose metabolism in three variants of primary progressive aphasia. Ann Neurol.

[70] Mesulam M, Wicklund A, Johnson N, Rogalski E, Léger GC, Rademaker A (2008). Alzheimer and frontotemporal pathology in subsets of primary progressive aphasia. Ann Neurol.

[71] Pereira JM, Williams GB, Acosta-Cabronero J, Pengas G, Spillantini MG, Xuereb JH (2009). Atrophy patterns in histologic vs clinical groupings of frontotemporal lobar degeneration. Neurology.

[72] Whitwell JL, Josephs KA, Rossor MN, Stevens JM, Revesz T, Holton JL (2005). Magnetic resonance imaging signatures of tissue pathology in frontotemporal dementia. Arch Neurol.

[73] Kim EJ, Rabinovici GD, Seeley WW, Halabi C, Shu H, Weiner MW (2007). Patterns of MRI atrophy in tau positive and ubiquitin positive frontotemporal lobar degeneration. J Neurol Neurosurg Psychiatry.

[74] Pickering-Brown SM, Richardson AM, Snowden JS, McDonagh AM, Burns A, Braude W (2002). Inherited frontotemporal dementia in nine British families associated with intronic mutations in the tau gene. Brain.

[75] Deramecourt V, Lebert F, Debachy B, Mackowiak-Cordoliani MA, Bombois S, Kerdraon O (2010). Prediction of pathology in primary progressive language and speech disorders. Neurology.

[76] Reed DA, Johnson NA, Thompson C, Weintraub S, Mesulam MM (2004). A clinical trial of bromocriptine for treatment of primary progressive aphasia. Ann Neurol.

[77] Decker DA, Heilman KM (2008). Steroid treatment of primary progressive aphasia. Arch Neurol.

[78] Boxer AL, Lipton AM, Womack K, Merrilees JR, Neuhaus J, Pavlic D (2009). An open-label study of memantine treatment in 3 subtypes of frontotemporal lobar degeneration. Alzheimer Dis Assoc Disord.

